# Real-world survival benefit of chemotherapy in elderly patients with advanced gastric cancer: a population-based SEER analysis

**DOI:** 10.1038/s41598-026-50084-2

**Published:** 2026-04-25

**Authors:** Nobuhiro Nakazawa, Kengo Kuriyama, Yuji Kumakura, Akiharu Kimura, Akihiko Sano, Makoto Sakai, Ken Shirabe, Hiroshi Saeki

**Affiliations:** Department of General Surgical Science, Gunma University Graduate School of Medicine, 3-39-22 Showa-machi, Maebashi, Gunma 371-8511 Japan

**Keywords:** Gastric cancer, Elderly, Chemotherapy, Survival, SEER, Cancer, Diseases, Gastroenterology, Medical research, Oncology

## Abstract

Using the U.S. Surveillance, Epidemiology, and End Results (SEER-21) database, we examined age-specific patterns of systemic chemotherapy utilization and survival outcomes among elderly and oldest-old patients with advanced gastric cancer (GC). Patients diagnosed with stage IV GC between 2004 and 2022 were stratified into four age groups (< 65, 65–74, 75–84, and ≥ 85 years). Clinicopathological characteristics and chemotherapy use were compared across age categories. Overall survival (OS) and cancer-specific survival (CSS) were evaluated using Kaplan–Meier analyses, and the association between chemotherapy and OS was assessed using propensity score-based inverse probability weighting and weighted Cox regression models. Among 56,478 eligible patients, chemotherapy utilization declined markedly with advancing age, from 69% in patients younger than 65 years to 20% in those aged 85 years or older. Chemotherapy was associated with significantly improved OS and CSS across all age groups (log-rank *P* < 0.0001). After adjustment, chemotherapy remained independently associated with improved OS in every age category, including patients aged 85 years or older (hazard ratio 0.40, 95% confidence interval 0.37–0.44). These findings demonstrate that systemic chemotherapy provides meaningful survival benefits even in the oldest-old population and underscore the importance of individualized, geriatric-informed treatment decision-making in advanced GC.

**Supplementary Information:**

The online version contains supplementary material available at 10.1038/s41598-026-50084-2.

## Introduction

Gastric cancer (GC) remains one of the most common gastrointestinal malignancies worldwide^1^. Although the age-standardized incidence rate (ASIR) and mortality of GC have declined in many developed countries owing to improved living conditions, dietary changes, and widespread *Helicobacter pylori* eradication programs, the proportion of elderly patients with GC has steadily increased. Globally, the burden of GC is strongly age-related, with incidence and mortality increasing substantially with advancing age, peaking in individuals aged ≥ 70 years. Although age-standardized incidence and mortality rates have generally declined across most regions from 1990 to 2019, older adults continue to experience the highest disease burden^2^. In high-income countries such as the United States, the highest age‑specific incidence rates of GC have been reported in the oldest age groups, with persons aged ≥ 85 years showing the greatest rates (60.65 per 100,000), followed by those aged 80–84 (57.55 per 100,000) and 75–79 years (47.83 per 100,000)^3^. Similarly, in East Asia, including Japan, the ASIR of GC declined between 1990 and 2019, with an average annual percent change of − 2.65% in men^4^. Despite this decline, the increasing number of elderly individuals has contributed to rising GC-related deaths. For example, in Japan, individuals aged ≥ 80 years accounted for approximately 49% (N = 21,596) of the 44,189 GC-related deaths in 2018, suggesting that rising mortality among the oldest age groups offsets the decline observed in younger populations^5^. These epidemiologic trends underscore the urgent need for diagnostic and therapeutic strategies tailored specifically to older and oldest-old patients with GC, who are often underrepresented in conventional clinical trials.

Elderly patients with GC exhibit distinct oncologic and clinical characteristics compared with younger individuals. Those aged ≥ 80 years are frequently diagnosed at advanced stages, often with impaired performance status (PS), and approximately one quarter present with Stage IV disease^6^. Treatment decisions in this group should prioritize biological frailty rather than chronological age^7^. However, evidence regarding the efficacy and safety of systemic chemotherapy in elderly patients remains limited^8^, as conventional clinical trials have largely underrepresented this group, leaving a substantial evidence gap.

Among the elderly, patients aged ≥ 85 years—hereafter referred to as the oldest-old—face even greater challenges. Data on the benefits and risks of systemic chemotherapy in this subgroup are scarce, and whether systemic chemotherapy meaningfully improves outcomes remains uncertain. Consequently, clear guidance regarding treatment eligibility or expected benefit is lacking, posing a frequent dilemma in real-world practice.

In evaluating the clinical characteristics and treatment outcomes of elderly patients with GC, the United States’ national cancer registry, the SEER (Surveillance, Epidemiology, and End Results) program, is highly valuable. SEER provides detailed information on patient age, sex, diagnostic stage, histology, treatment modalities, and survival outcomes, with long-term follow-up, making it widely used for epidemiologic studies and survival analyses^9^. Notably, elderly and oldest-old patients are often underrepresented in conventional clinical trials, and analyses using SEER can help elucidate real-world treatment patterns and prognostic trends in these populations. Thus, SEER is an important data source for predicting outcomes and guiding systemic chemotherapy in elderly GC patients.

In the present study, we aimed to investigate the real-world clinical use of systemic chemotherapy in elderly and oldest-old patients with advanced GC. We also assessed whether chemotherapy confers a survival benefit compared with patients who did not receive such treatment. Using the U.S. national cancer registry, the SEER program, we analyzed chemotherapy utilization rates, survival outcomes, and associated factors across different age groups. This approach allowed us to evaluate the effectiveness and prognostic impact of systemic chemotherapy in populations often underrepresented in conventional clinical trials, thereby providing essential insights to guide evidence-based treatment strategies and future guideline development for elderly GC patients.

## Results

### Patient characteristics

Patient characteristics are summarized by age group (< 65, 65–74, 75–84, and ≥ 85 years) in Table 1. A total of 56,478 patients were included, comprising 26,662 (< 65), 14,742 (65–74), 11,241 (75–84), and 3,833 (≥ 85) patients in each respective age category.

**Table 1:** Baseline patient characteristics stratified by age group.

Patient characteristics	Age group			
	< 65	65–74	75–84	≧ 85
	N = 26,662	N = 14,742	N = 11,241	N = 3833
Sex				
Male	16,820 (63%)	9894 (67%)	7057 (63%)	2073 (54%)
Female	9842 (37%)	4848 (33%)	4184 (37%)	1760 (46%)
Years of diagnosis				
2004–2008	6131 (23%)	3276 (22%)	2873 (26%)	889 (23%)
2009–2013	6895 (26%)	3506 (24%)	2907 (26%)	1012 (26%)
2014–2018	7462 (28%)	4193 (28%)	2987 (27%)	1075 (28%)
2019–2022	6174 (23%)	3767 (26%)	2474 (22%)	857 (22%)
Race				
White	19,490 (74%)	10,999 (75%)	8456 (75%)	2887 (76%)
Black	3763 (14%)	2014 (14%)	1474 (13%)	453 (12%)
American Indian/Alaska Native	295 (1.1%)	107 (0.7%)	64 (0.6%)	12 (0.3%)
Asian or Pacific Islander	2968 (11%)	1561 (11%)	1217 (11%)	470 (12%)
Median household income				
Low (< 60,000 USD)	3555 (13%)	2107 (14%)	1537 (14%)	460 (12%)
Middle (60,000–99,999 USD)	18,069 (68%)	9586 (65%)	7352 (65%)	2458 (64%)
High (≧ 100,000 USD)	5037 (19%)	3048 (21%)	2352 (21%)	915 (24%)
Urbanicity				
Non-metro	2429 (9%)	1635 (11%)	1180 (11%)	335 (9%)
Metro-small	6192 (23%)	3582 (24%)	2753 (25%)	936 (24%)
Metro-large	17,940 (68%)	9486 (65%)	7297 (65%)	2560 (67%)
Tumor location				
Cardia	8196 (31%)	5277 (36%)	3279 (29%)	907 (24%)
Fundus/body	3901 (15%)	1995 (14%)	1704 (15%)	666 (17%)
Antrum/pylorus	4154 (16%)	2357 (16%)	2128 (19%)	872 (23%)
Other/overlapping	10,411 (39%)	5113 (35%)	4130 (37%)	1388 (36%)
Tumor differentiation				
Well differentiated	223 (1.5%)	189 (2.4%)	178 (2.8%)	83 (3.8%)
Moderately differentiated	2732 (18%)	1969 (25%)	1722 (27%)	631 (29%)
Poorly/undifferentiated	12,067 (80%)	5857 (73%)	4561 (71%)	1449 (67%)
Operation				
Absent	19,399 (86%)	10,866 (87%)	8041 (88%)	2823 (90%)
Present	3192 (14%)	1610 (13%)	1142 (12%)	309 (10%)
Chemotherapy				
No/Unknown	8156 (31%)	6014 (41%)	6419 (57%)	3053 (80%)
Yes	18,506 (69%)	8728 (59%)	4822 (43%)	780 (20%)

Overall, sex distribution was relatively balanced, with males representing 54–67% and females 33–46% across age groups. Year of diagnosis was evenly distributed across the four periods (2004–2008, 2009–2013, 2014–2018, and 2019–2022), each accounting for approximately 22–28% of cases per age group. Racial composition was largely consistent, with the majority being White (74–76%), followed by Black (12–14%), Asian or Pacific Islander (11–12%), and American Indian/Alaska Native (0.3–1.1%). Socioeconomic status, assessed by median household income, was broadly similar across age groups, with the majority of patients falling in the middle-income category (60,000–99,999 USD). Urbanicity distribution was also comparable, with 65–68% of patients residing in large metropolitan areas.

Tumor location exhibited slight age-related variation: Cardia tumors were most frequent among patients aged 65–74 years (36%), whereas the proportion of Antrum/Pylorus tumors increased with age, reaching 23% in patients ≥ 85 years. Tumor differentiation tended to improve with age, with poorly or undifferentiated tumors being relatively more frequent in younger patients (< 65: 80%) than in the oldest-old (≥ 85: 67%).

**Fig. 1:**
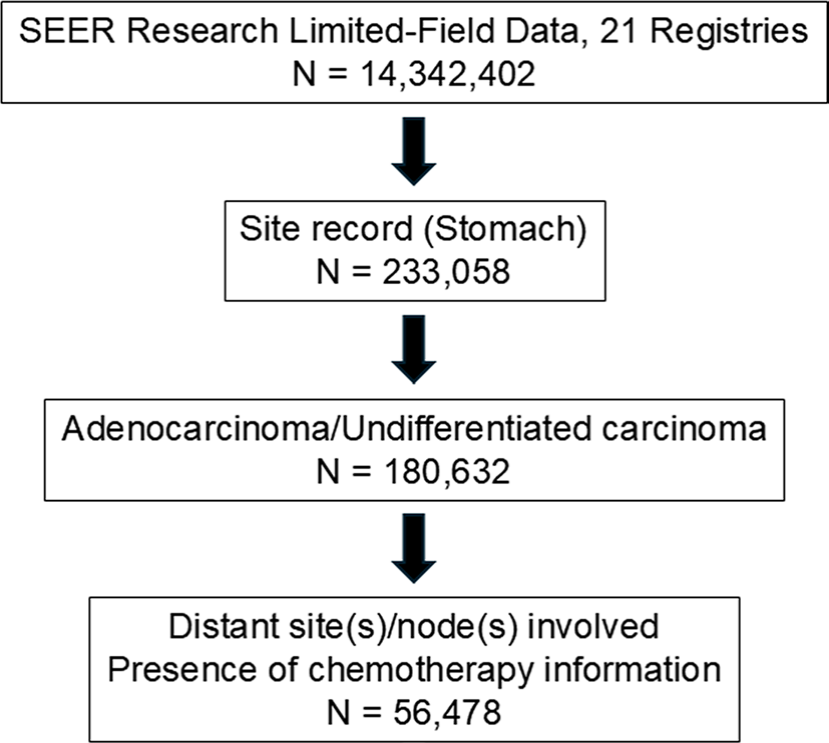
Study flowchart for patient selection.

Surgical intervention was performed in a minority of patients, with rates slightly declining with age (< 65: 14%, ≥ 85: 10%). In contrast, chemotherapy utilization decreased markedly with age, from 69% in patients < 65 years to 20% in those ≥ 85 years, although within each age group, chemotherapy utilization showed a modest increase over successive diagnosis periods (Fig. 1).

### Survival analysis

**Fig. 2:**
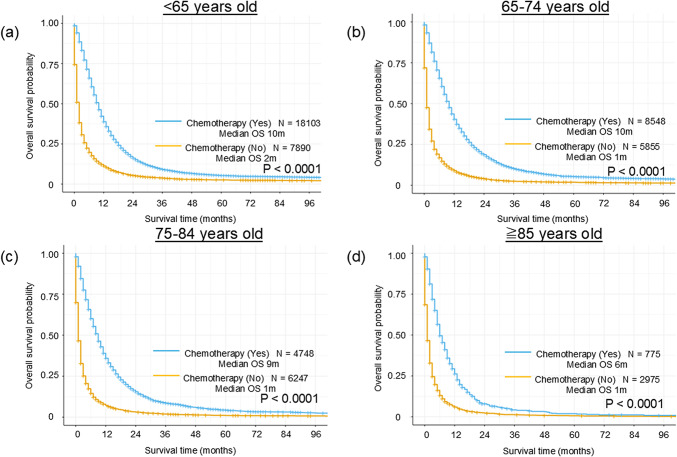
Overall survival stratified by age and treatment.

**Fig. 3:**
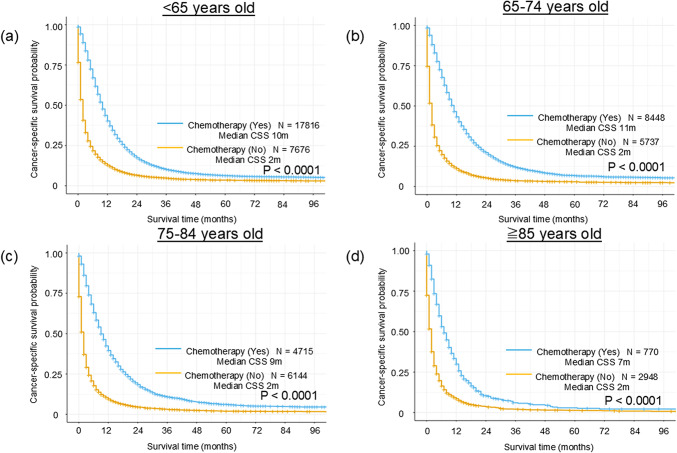
Cancer-specific survival stratified by age and treatment.

OS stratified by age groups (< 65, 65–74, 75–84, and ≥ 85 years) is shown in Fig. 2. In all age categories, chemotherapy was associated with significantly improved OS (*P* < 0.0001). Comparable findings were observed for CSS (Fig. 3).

### Effect of chemotherapy on OS across age groups

The impact of chemotherapy on OS was evaluated across four age groups (< 65, 65–74, 75–84, and ≥ 85 years) using inverse probability weighting (IPW)-adjusted Cox proportional hazards models (Table 2). Chemotherapy was associated with a marked reduction in the risk of death in all age categories. Specifically, the HRs for patients receiving chemotherapy were 0.39 (95% CI 0.38–0.41) for those < 65 years, 0.34 (95% CI 0.32–0.36) for 65–74 years, 0.34 (95% CI 0.33–0.36) for 75–84 years, and 0.40 (95% CI 0.37–0.44) for ≥ 85 years, all with *P* < 0.0001. These findings indicate that chemotherapy confers a substantial survival benefit regardless of patient age.

**Table 2:** Effect of chemotherapy on overall survival stratified by age group (inverse probability weighting-adjusted Cox model).

Age group	No. of cases	No. of events	HR (95% CI)	*P* value
< 65	22,133	19,415	0.39 (0.38–0.41)	< 0.0001
65–74	12,221	10,903	0.34 (0.32–0.36)	< 0.0001
75–84	9022	8318	0.34 (0.33–0.36)	< 0.0001
≥ 85	3075	2956	0.40 (0.37–0.44)	< 0.0001

CI, confidence interval; HR, hazard ratio.

## Discussion

Given the rapidly ageing populations worldwide, disparities in treatment utilization and survival among patients with advanced gastric cancer (GC) are of increasing global relevance. In this study, we aimed to investigate the real-world utilization and effectiveness of systemic chemotherapy in elderly and oldest-old patients with advanced GC, a population largely underrepresented in conventional clinical trials. Using the U.S. SEER national cancer registry, we evaluated chemotherapy administration patterns, OS, and CSS across four age groups (< 65, 65–74, 75–84, and ≥ 85 years). Our findings further demonstrate that chemotherapy was consistently associated with improved survival across age groups, including in the oldest-old (≥ 85 years), as shown in IPW-adjusted analyses for OS. These results provide population-level evidence suggesting a potential survival advantage associated with systemic chemotherapy in elderly patients with advanced GC and highlight the potential for improving outcomes in age groups traditionally considered at high risk or undertreated.

In a retrospective study, Kim et al. evaluated the clinical impact of palliative chemotherapy in patients with advanced GC. Their findings indicated that chemotherapy contributed to maintaining quality of life (QOL), and that OS and CSS among patients receiving first-line chemotherapy did not differ significantly between age groups stratified at 70 years^10^. Nevertheless, older patients tended to receive less intensive regimens, reflecting cautious or suboptimal treatment practices in real-world settings. Age-related comorbidities and changes in pharmacokinetics and pharmacodynamics may increase toxicity, often making oncologists hesitant to administer systemic chemotherapy to elderly patients^8^. Consistent with these reports, our data demonstrated an age-dependent decline in chemotherapy administration: 69% in patients < 65, 59% in those aged 65–74, 43% in patients aged 75–84, and only 20% in patients ≥ 85 years.

Large-scale, real-world evidence assessing the prognostic benefit of chemotherapy in older adults remains limited, which was one of the primary objectives of this study. Current guidelines emphasize the incorporation of geriatric assessment (GA) into treatment decision-making, as such evaluations provide clinically relevant information beyond chronological age alone^11–13^. Comprehensive geriatric assessment (CGA), which includes functional status, comorbidities, nutritional and cognitive domains, has been shown to complement conventional performance status when considering chemotherapy in elderly patients^14,15^. In our cohort, although chemotherapy was associated with improved survival even among the oldest-old (≥ 85 years), only 20% of these patients received systemic treatment, indicating that chemotherapy is not appropriate for all older adults. In GC, screening tools such as the Geriatric 8 (G8) may help identify elderly patients who could tolerate and potentially benefit from first-line chemotherapy^16,17^. These findings highlight the importance of individualized, geriatric-guided treatment strategies and the need for further prospective validation.

In elderly patients with GC, tumor differentiation and location exhibit age-dependent patterns that may influence both chemotherapy response and prognosis. Specifically, younger patients tend to develop poorly or undifferentiated tumors, whereas the proportion of relatively well-differentiated tumors increases in the oldest age group (≥ 85 years). Regarding tumor location, cardia tumors are more common in middle-aged to early elderly patients (65–74 years), while tumors in the antrum/pylorus region show an increasing trend with advancing age. These age-related differences in tumor characteristics may affect tumor aggressiveness, growth rate, and sensitivity to chemotherapy. In younger patients, aggressive and rapidly progressing tumors often prompt intensive surgical resection and chemotherapy; however, such interventions do not necessarily translate into improved outcomes^18,19^. In contrast, selected elderly patients may achieve favorable responses with tailored chemotherapy regimens, including dose modification or fractionated administration^20–22^. Consideration of age-related tumor biology may therefore support more individualized treatment strategies across age groups.

Another important consideration in elderly patients with advanced GC is the potential impact of systemic chemotherapy on QOL. In the palliative setting, treatment goals often include symptom control and maintenance of QOL in addition to survival prolongation. Previous studies have suggested that systemic chemotherapy may maintain or improve QOL in selected patients with advanced GC, although treatment-related toxicities may adversely affect patient-reported outcomes depending on regimen intensity and patient fitness^23,24^. In addition, treatment refusal may partly contribute to the lower chemotherapy utilization observed in older age groups. Elderly patients may decline systemic therapy because of concerns regarding toxicity, comorbidities, or perceived limited benefit. Unfortunately, the SEER database does not capture information on patient treatment preferences or reasons for non-treatment. Therefore, we were unable to determine whether treatment refusal differed across age groups in the present study.

The strengths of this study include the use of the U.S. SEER national cancer registry, which allowed evaluation of chemotherapy utilization and survival outcomes in elderly and oldest-old patients with advanced GC using a large, population-representative real-world dataset. The application of IPW enabled statistical balancing of measured covariates, mitigating potential confounding inherent in observational studies and approximating conditions similar to a randomized controlled trial. Moreover, the detailed age-stratified analysis allowed assessment of whether systemic chemotherapy confers a survival benefit even among the oldest-old, enhancing the clinical relevance and novelty of our findings. Nevertheless, several limitations should be acknowledged. First, the SEER database does not include detailed clinical variables such as performance status, frailty status, or comorbidity indices (e.g., Charlson Comorbidity Index). Consequently, the observed survival benefit associated with chemotherapy may partly reflect selection bias, whereby clinicians preferentially administer systemic therapy to fitter elderly patients. Although the use of IPW reduced confounding from measured variables, residual confounding due to unmeasured clinical factors cannot be excluded. Second, SEER records systemic chemotherapy as a binary variable and does not provide detailed information on treatment regimens, dosages, treatment intensity, or treatment-related toxicities. Therefore, we were unable to evaluate regimen-specific efficacy or treatment tolerability, which is particularly important in elderly and oldest-old populations. Third, the treatment landscape for advanced gastric cancer has evolved substantially in recent years with the introduction of immune checkpoint inhibitors and targeted therapies. Because the SEER database does not capture detailed information on these agents, our analysis could not account for potential differences in modern treatment strategies. Fourth, as with all observational studies using registry data, causal inference regarding the effect of chemotherapy should be interpreted with caution. Finally, because information on the exact initiation date of chemotherapy was not available in our SEER dataset, we could not perform a time-dependent analysis, and some residual immortal time bias may remain, potentially leading to overestimation of the observed survival benefit of chemotherapy.

In conclusion, our findings demonstrate that systemic chemotherapy is associated with meaningful survival advantages even among the oldest-old patients with advanced GC. Despite markedly lower treatment utilization in this population, appropriately selected elderly individuals can still derive substantial benefit, emphasizing the importance of individualized treatment decisions guided by biological rather than chronological age. As populations continue to age globally, integrating geriatric assessment into clinical practice and tailoring chemotherapy strategies to the unique physiological and tumor characteristics of older adults will be essential to achieving equitable and effective cancer care across all age groups.

## Methods

### Study population

We used data from the National Cancer Institute’s SEER Research Limited-Field Data, 21 registries (excl IL), November 2024 submission, covering cases diagnosed from 2000 to 2022 (N = 14,342,402). This database captures approximately 42% of the U.S. population and provides reliable information on cancer incidence and survival^25^. Patients with stage IV GC were identified from this dataset, as outlined in the flowchart (Fig. 3). Case selection was based on the Site Recode International Classification of Diseases for Oncology, 3rd Edition (ICD-O-3)/WHO 2023 Revision for stomach as the primary site (N = 233,058). Histologic subtypes were limited to adenocarcinoma with variants of stomach (code 8.1) and undifferentiated carcinoma of stomach (code 8.4) according to the Site Recode—Rare Tumors classification (N = 180,632). Disease stage was defined using the Combined Summary Stage with Expanded Regional Codes (2004+), and only cases classified as distant site(s)/node(s) involved and having complete information on chemotherapy status were included (N = 56,478).

### Clinicopathological characteristics

SEER*Stat version 9.0.41.4 was used to extract data for descriptive analysis. The following clinicopathological variables were extracted from the dataset: sex (male vs. female), year of diagnosis (2004–2008, 2009–2013, 2014–2018, 2019–2022), race/ethnicity (White, Black, American Indian/Alaska Native, Asian or Pacific Islander), tumor location (cardia, fundus/body, antrum/pylorus, other/overlapping), tumor differentiation (well-differentiated, moderately differentiated, poorly differentiated/undifferentiated), surgical treatment (absent vs. present), chemotherapy (no/unknown vs. yes), median household income (low: < 60,000 USD, median: 60,000–99,999 USD, high: ≥ 100,000 USD), and urbanicity (non-metropolitan, small metropolitan, large metropolitan). Patients were stratified by age into four groups: < 65, 65–74, 75–84, and ≥ 85 years, and analyses were performed to evaluate the real-world use of systemic chemotherapy in elderly and oldest-old patients with advanced GC.

### Statistical analysis

Overall survival (OS) and cancer-specific survival (CSS) were estimated using the Kaplan–Meier method, and survival curves were compared with the log-rank test. Only patients with complete survival time information were included in the analysis, as determined by the Survival months flag variable. OS was defined as the time from diagnosis to death from any cause, and CSS was defined as the time from diagnosis to death attributable to GC. For subgroup analyses, patients were stratified according to age group (< 65, 65–74, 75–84, and ≥ 85 years) and receipt of systemic chemotherapy (yes vs. no/unknown).To address potential confounding in the analysis of OS, propensity scores for receipt of systemic chemotherapy were estimated using a multivariable logistic regression model including demographic factors (sex, year of diagnosis, race), tumor characteristics (tumor location, tumor differentiation), treatment factors (surgery status), and socioeconomic factors (median household income, rural–urban residence). Age was not included in the propensity score model because the primary aim was to allow evaluation of age-specific chemotherapy effects without controlling away age differences. Inverse probability of weighting (IPW) was then applied to balance the measured covariates between treatment groups, generating a weighted pseudo-population, which is a statistical construct that balances baseline characteristics between groups to mimic a randomized trial. Covariate balance was assessed using standardized mean differences (SMDs), with values below 0.1 indicating adequate balance (Supplementary Table 1). Weighted Cox proportional hazards models were subsequently used to estimate hazard ratios (HRs) and 95% confidence intervals (CIs) for the effect of chemotherapy on OS, with interaction terms included to evaluate whether the effect of chemotherapy differed across age groups.

All statistical analyses were conducted using R software (version 4.5.1). All *P* values were two-sided, and statistical significance was set at a stringent level of α = 0.005, as recommended by expert consensus^26^.

## Supplementary Information

Below is the link to the electronic supplementary material.

Supplementary Material 1

## Data Availability

The data analyzed in this study are publicly available from the SEER database (https://seer.cancer.gov) after signing a data-use agreement. Derived datasets and analysis codes are available from the corresponding author upon reasonable request.

## Abbreviations

ASIR: Age-standardized incidence rate
CGA: Comprehensive geriatric assessment
CI: Confidence interval
CSS: Cancer-specific survival
GA: Geriatric assessment
GC: Gastric cancer
G8: Geriatric 8
HR: Hazard ratio
ICD-O-3: International Classification of Diseases for Oncology, 3rd Edition
IPW: Inverse probability of weighting
OS: Overall survival
PS: Performance status
QOL: Quality of life
SEER: Surveillance, Epidemiology, and End Results
SMD: Standardized mean difference
