# Supplementary material for: Real-world survival benefit of chemotherapy in elderly patients with advanced gastric cancer: a population-based SEER analysis
Source: Sci Rep. 2026 Apr 25;16:19126. doi: 10.1038/s41598-026-50084-2 (PMC13280379; doi:10.1038/s41598-026-50084-2)
Supplement: Supplementary file 1 — Supplementary Material 1 [file 41598_2026_50084_MOESM1_ESM.docx]

Supplementary Table 1. Covariate balance before and after inverse probability weighting.

|  | SMD_Unweighted | SMD_IPW |
| --- | --- | --- |
| Sex | 0.071 | 0.004 |
| Year of diagnosis | 0.163 | -0.001 |
| Race | 0.111 | 0.002 |
| Tumor location | 0.250 | 0.001 |
| Tumor differentiation | 0.113 | 0.002 |
| Surgery performed | 0.065 | 0.012 |
| Median household income | 0.098 | 0.007 |
| Urbanicity | 0.051 | 0.007 |

Abbreviations: IPW, inverse probability weighting; SMD, standardized mean differences.
